# Integrating a Mobile Health Device Into a Community Youth Mental Health Team to Manage Severe Mental Illness: Protocol for a Randomized Controlled Trial

**DOI:** 10.2196/19510

**Published:** 2020-11-02

**Authors:** Simon Byrne, Beth Kotze, Fabio Ramos, Achim Casties, Jean Starling, Anthony Harris

**Affiliations:** 1 Western Sydney Local Health District Mental Health Service Sydney, NSW Australia; 2 Westmead Institute for Medical Research Sydney Australia; 3 Rivendell Child Adolescent and Family Unit Sydney Australia; 4 School of Computer Science University of Sydney Sydney Australia; 5 Concord Centre for Mental Health Sydney Australia; 6 Discipline of Psychiatry Sydney Medical School University of Sydney Sydney Australia

**Keywords:** electrodermal activity, anxiety, psychosis, mHealth device, actigraphy

## Abstract

**Background:**

Symptoms of mental illness are often triggered by stress, and individuals with mental illness are sensitive to these effects. The development of mobile health (mHealth) devices allows continuous recording of biometrics associated with activity, sleep, and arousal. Deviations in these measures could indicate a stressed state requiring early intervention. This paper describes a protocol for integrating an mHealth device into a community mental health team to enhance management of severe mental illness in young adults.

**Objective:**

The aim of this study is to examine (1) whether an mHealth device integrated into a community mental health team can improve outcomes for young adults with severe mental illness and (2) whether the device detects periods of mental health versus deterioration.

**Methods:**

This study examines whether physiological information from an mHealth device prevents mental deterioration when shared with the participant and clinical team versus with the participant alone. A randomized controlled trial (RCT) will allocate 126 young adults from community mental health services for 6 months to standard case management combined with an integrated mHealth device (ie, physiological information is viewed by both participant and case manager: unWIRED intervention) or an unintegrated mHealth device (ie, participant alone self-monitors: control). Participants will wear the Empatica Embrace2 device, which continuously records electrodermal activity and actigraphy (ie, rest and activity). The study also examines whether the Embrace2 can detect periods of mental health versus deterioration. A variety of measurements will be taken, including physiological data from the Embrace2; participant and case manager self-report regarding symptoms, functioning, and quality of life; chart reviews; and ecological momentary assessments of stress in real time. Changes in each participant’s Clinical Global Impression Scale scores will be assessed by blinded raters as the primary outcome. In addition, participants and case managers will provide qualitative data regarding their experience with the integrated mHealth device, which will be thematically analyzed.

**Results:**

The study has received ethical approval from the Western Sydney Local Health District Human Research Ethics Committee. It is due to start in October 2020 and conclude in October 2022.

**Conclusions:**

The RCT will provide insight as to whether an integrated mHealth device enables case managers and participants to pre-emptively manage early warning signs and prevent relapse. We anticipate that unWIRED will enhance early intervention by improving detection of stress and allowing case managers and patients to better engage and respond to symptoms.

**Trial Registration:**

Australian New Zealand Clinical Trials Registry (ANZCTR) ACTRN12620000642987; https://www.anzctr.org.au/ACTRN12620000642987.aspx

**International Registered Report Identifier (IRRID):**

PRR1-10.2196/19510

## Introduction

### Overview

Severe mental illnesses, such as schizophrenia, bipolar disorder, and borderline personality disorder are associated with considerable burden and have their peak onset during early adulthood [1,2]. These disorders are highly disruptive in terms of functioning, impairing a young adult’s ability to maintain close relationships, educate themselves, and find employment [1,3]. For example, youths at risk of psychotic illnesses often experience significant functional decline alongside the onset of symptoms [4,5]. The extent to which a mental illness prevents functioning is often the threshold for a psychiatric diagnosis [6,7]. Severe mental illness is also associated with high levels of distress and high rates of suicide compared to the general population [8]. While there is typically no cure for severe mental illness, symptoms can be managed in the community with combinations of medications and/or psychosocial treatments [9,10]. Effective case management requires collaboration between patient and clinician, as well as rapid identification and treatment of early warning signs [3,11]. Yet case management faces several challenges, including patients having limited insight into emerging symptoms and case managers not being able to respond promptly [3,9]. Even small delays prior to intervention could mean the difference between a minor relapse and a lengthy hospitalization.

A potential means to enhance early intervention and engagement is through the use of an inexpensive mobile health (mHealth) device that measures physiological indicators of stress and returns this information to patients and case managers. Across the spectrum of mental disorders, elevated stress levels are known to increase risk of relapse. The body and mind have systems that maintain a homeostatic state and these can be disrupted by acute or chronic stress [12,13]. For example, stress can impact upon the autonomic nervous system and circadian rhythms, causing changes in arousal and sleep patterns, respectively. While small amounts of stress can be beneficial, acute or prolonged stress is harmful, and individuals with mental illness are particularly sensitive to these effects. An mHealth device can be worn like a wristwatch that can reliably and continuously measure physiological signs of stress. For example, mHealth devices can measure arousal through electrodermal activity (EDA). An mHealth device can also measure actigraphy, which is a noninvasive method of monitoring human rest and activity cycles [14]. A small actigraphy unit has an accelerometer that measures gross motor activity across three axes. These measurements can be converted into variables of interest, such as step counts and periods of rest. Changes in these measures may indicate an individual is experiencing a level of stress that could be harmful. For example, a combination of high arousal and poor sleep suggests a stress response associated with greater risk of relapse. Less activity could be associated with a depressive state and greater levels could be associated with a manic state [15]. Physiological data from an mHealth device can also be combined with other related variables to predict relapse [15,16]. For example, an ecological momentary assessment (EMA) [17] involves taking subjective measurements of an individual’s current mental state, which can be used in conjunction with physiological data from an mHealth device.

mHealth devices have been used effectively for managing and monitoring physical conditions such as cardiovascular disorders, diabetes, and obesity [18]. More recently, continuous data from an mHealth device has shown promise in predicting signs of stress and mental deterioration. For example, Sano and colleagues [19] examined whether physiological and behavioral measures collected by wearable sensors and mobile phones could predict stress and poor mental health in a large student sample (N=201). They used machine learning, a subfield of artificial intelligence, to analyze the multimodal data. The mHealth devices classified students into high- or low-stress groups 78.3% of the time and as experiencing high or low mental health 87% of the time. Cella and colleagues [20] used an mHealth device to compare heart rate, EDA, and movement for 30 participants with schizophrenia in the community and 25 controls. Compared to controls, participants with schizophrenia showed lower heart rate variability, movement, and functioning, consistent with autonomic dysregulation. Positive symptoms were also correlated with parasympathetic dysregulation. Most recently, Cella and colleagues [21] asked participants with first episode psychosis to wear an mHealth device to continuously measure their heart rate variability and EDA while self-reporting psychotic symptoms. They found higher EDA during periods where participants were experiencing psychosis.

**Figure 1:**
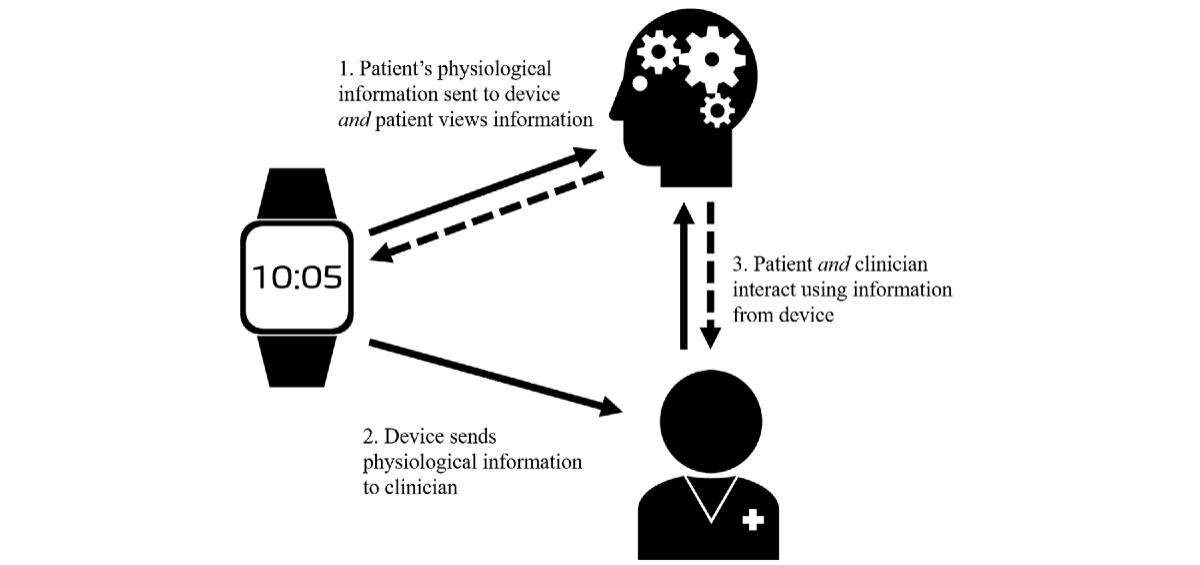
Information flow between patient, clinician, and integrated mobile health device.

The use of an mHealth device in younger adults with mental illness over extended periods has yet to be tested. This age group is more likely to be “tech savvy” and they are at high risk of developing severe mental illness. Pilot data indicate 15 out of 19 young adults with severe mental illness wore an mHealth device for greater than two weeks, and information regarding each participant’s activity, sleep, EDA, and subjective stress was reliably collected during this period [22]. The next challenge is establishing if this information is clinically useful and predicts relapse. This protocol describes using information from an mHealth device streamed to both case managers and patients to allow them to independently monitor and then collaborate to manage any early warning signs (see Figure 1).

### Aims and Hypotheses

The aims of this study are to (1) examine whether case management augmented by an mHealth device integrated into a youth mental health team can improve treatment outcomes for young people with a severe mental illness and (2) examine whether the mHealth device can predict mental deterioration versus health for those allocated to the integrated arm of the trial. We hypothesize that (1) case management for a severe mental illness enhanced by an mHealth device integrated into a youth mental health team will result in better outcomes than case management with an unintegrated device and (2) physiological data from an mHealth device will identify periods of mental deterioration.

## Methods

### Overview

The trial design is a parallel-group randomized controlled trial (RCT) predicting the superiority of case management for severe mental illness with an integrated mHealth device over case management with an unintegrated device. It will examine outcomes both between subjects (ie, case management with an integrated vs unintegrated mHealth device) and within subjects (ie, repeated measures of participants with the integrated device to determine if it predicts mental deterioration). The conditions vary regarding whether information from the mHealth device is shared with participants and clinicians *or* with participants alone. Participants will be randomly allocated to either (1) the integrated (unWIRED) condition, where information from the mHealth device is shared with both participant and clinician, or (2) the unintegrated condition, where information from the mHealth device is sent to the participant alone. Participation in the study will last for 6 months. The design and reporting of this study will follow the CONSORT-EHEALTH (Consolidated Standards of Reporting Trials of Electronic and Mobile Health Applications and Online Telehealth) guidelines [23]. This protocol was registered with the Australian New Zealand Clinical Trials Registry (ANZCTR) (ACTRN12620000642987).

### Participants

The study will recruit 126 youths aged 18-25 years with a severe mental illness drawn from youth community mental health services in Western Sydney and Sydney Local Health Districts in New South Wales (NSW), Australia, where BK, JS, and AH are senior psychiatrists. Admission to these services is determined by participants experiencing moderate to severe mental illness that impairs functioning. All participants at these sites will be receiving case management for their mental illness, including regular medication monitoring and/or psychosocial treatments consistent with Australian and New Zealand College of Psychiatry guidelines. Participants will not be paid; however, they can be reimbursed for costs associated with participation, such as travel expenses or mobile phone credit.

Inclusion criteria are (1) being a current community mental health service patient, (2) aged 18-25 years, (3) having a confirmed diagnosis of serious mental illness, including schizophrenia spectrum disorders, bipolar affective disorder, severe major depressive disorder, anxiety disorder, and/or personality disorder, and (4) having the capacity to consent to the study.

Exclusion criteria are (1) being non-English speaking, (2) having greater than mild developmental disability, and (3) having an inability to comply with either the requirements of informed consent or the treatment protocol.

Substance use will not be an exclusion criterion, as the effects of stress due to substance withdrawal and self-medication are important factors in relapse. It is also important to keep the study population ecologically similar to the clinical population.

### Equipment

The Empatica Embrace2 [24] will be used as the mHealth device in the study. The Embrace2 is an mHealth device that can be worn like a wristwatch. It has a wearable biosensor that captures, stores, and wirelessly transmits EDA and motion data. The Embrace2 is paired to a mobile phone via Bluetooth, where information is displayed on the Mate App (Empatica Inc) of the participant’s phone. The Mate App is a free app developed for the Embrace2 device. Data from the Embrace2 are uploaded to a secure Empatica cloud server. The device will be worn continuously to monitor physiological state, day and night. It requires charging every 48 hours using a USB and dock. In addition to the Embrace2, the young person will need to own a compatible mobile phone that runs on either the Apple iOS or Android operating system. Basic Android phones will be lent to participants who do not have a suitable phone. The mobile phone will have the free Mate App downloaded for the Embrace2 device. The phone can display activity-related information to the participant through the app. The Mate App also allows participants to log *events*, or make digital diary entries, that can be time stamped against their physiological data.

### Interventions

#### Overview

All participants will receive standard treatments, including case management for their severe mental illness. This involves assessment followed by combinations of medication-based and/or psychosocial treatments. There is a strong emphasis on early intervention in these services. Prospective participants are initially assessed by a multidisciplinary team before being admitted according to standard inclusion criteria. The main criterion for admission is that they are experiencing a moderate to severe mental illness that impairs functioning. Participants are assigned a core case manager; however, their treatment is managed within a team of psychiatrists, nurses, psychologists, occupational therapists, and/or social workers. Treatments offered include case management, medication, psychotherapy, cognitive remediation, vocational interventions, and group programs (eg, psychoeducation). After consenting to the trial, participants at these services will be randomly allocated to one of two interventions that vary with respect to who receives information from the mHealth device.

#### Integrated mHealth Device: unWIRED Intervention

Participants will receive case management for their mental illness as well as wearing the Embrace2 device. This device sends physiological measurements to the Empatica Research Portal, which can then be relayed to health service computers for the treating case manager to view. A dashboard generated by the program Splunk [25] provides case managers with information regarding each participant’s arousal, sleep, and activity from the previous day. Splunk captures, indexes, and correlates real-time data and generates graphs, reports, dashboards, and visualizations. The dashboard provides a visualization of the participant’s EDA and activity over a 24-hour period, as well as representing activity through *speed meters*, which present the participant’s level of activity. The program provides password-protected weblinks allowing clinical staff to view the dashboard from their clinical site. Each participant will also have access to activity-based information, which will be transmitted to their mobile phone’s Mate App. Based on the information provided, case managers and/or participants may choose to alter their management plan. For example, periods of high arousal and/or poor sleep may indicate a need for a medication change or a more assertive intervention. Continuous data will be visually inspected for significant deviations in physiological measures and the *speed meters* will be used to alert clinicians of significant change. Physiological information relayed to both the case manager and participant will be regularly reviewed and discussed during team meetings. Where possible, the use of the dashboard will be recorded in electronic medical records. Adherence to the device will be monitored through a portal that shows if the device is being worn and the data are streaming. If there are consecutive days of nonadherence, the research psychologist (SB) will contact the participant to find out the reason for the disconnection and attempt to troubleshoot. For example, the psychologist may ask participants to remember to wear or charge the device and insure it is paired with their phone.

#### Unintegrated mHealth Device: Control Condition

Participants in the control condition will receive their standard care in addition to being given an Embrace2 to self-monitor their activity levels. This device will provide information to each participant regarding their activity; however, unlike in the integrated condition, information will not be returned to their treating case manager—the device will not be integrated with the mental health team. The device can be used at each participant’s discretion, without prompting or oversight from their case manager.

### Randomization

Randomization will be conducted by an independent statistician not associated with the study. Randomly permuted blocks of 4 participants will be centrally generated using a computer-generated algorithm. The randomization sequence will be managed by staff independent of the project who will notify the clinical researcher of the randomization allocation. Participants, case managers, and clinician researchers at any one site will not be blinded as to allocation, as those in the integrated condition will regularly discuss the intervention with their case manager.

### Measurements

#### Overview

The primary outcome for the RCT is the extent to which the integrated mHealth device can prevent mental deterioration. The within-subjects components of the trial will examine if differences in activity, sleep, and EDA predict mental deterioration versus stability. A variety of measurements will be taken, including self-report, case manager report, biometric measures from the Embrace2, EMAs of stress, chart reviews, as well as qualitative reports from case managers and participants regarding the acceptability of the device. Multimedia Appendix 1 shows a summary of the measurements taken at each time point.

#### Assessments of Mental Deterioration

Case managers and blinded clinical staff will make monthly assessments of participants using the Clinical Global Impression Scale (CGI) [26], the Social and Occupational Functioning Assessment Scale (SOFAS) [6], and chart reviews.

##### The Clinical Global Impression Scale

The primary measures are consensus scores on the CGI severity and treatment response scales. Assessments are made by blinded clinicians, taking into account all relevant information to make ratings on two 7-point scales. The CGI is a brief, valid measure of symptom severity and treatment response, which has shown utility in measuring mental state as compared to similar scales [27,28].

##### The Social and Occupational Functioning Assessment Scale

The SOFAS is an overall measure of social and occupational functioning used in psychiatric assessments. The SOFAS describes functioning of the participant at the time of the assessment. Its ratings are based on a 0-100 scale, reflecting excellent to grossly impaired functioning, with higher scores indicating better functioning.

##### Chart Reviews

Clinical research staff will primarily conduct chart reviews using electronic medical records to make CGI and SOFAS assessments of participants in the trial. Charts will be examined for information including diagnosis, medication histories, and occasions of service (ie, treatment-as-usual information); the period between emergence of symptoms and intervention; evidence of illicit substance use; hospitalizations; indications of relapse; and evidence of case manager use of the portal.

#### Participant Self-Report, With Time Point

##### Demographics

Demographic information includes gender, date of birth, and ethnicity and is collected at baseline only.

##### The 10-Item Big Five Inventory

The 10-item Big Five Inventory (BFI-10) [29] is a personality measure based on the 44-item Big Five Inventory [30]. The BFI-10 focuses on five broad personality domains, including openness, conscientiousness, neuroticism, extraversion, and agreeableness. It is rated on a 5-point Likert scale, ranging from 1 (strongly disagree) to 5 (strongly agree). The BFI-10 retained validity compared to the original version and its test-retest reliability was .75. This inventory is administered at baseline only.

##### The 21-Item Depression Anxiety and Stress Scale

The 21-item Depression Anxiety and Stress Scale (DASS-21) [31] is a measure of depression, anxiety, and stress over the previous week. Respondents rate each item on a 4-point Likert scale, ranging from 0 (not at all) to 3 (almost always). The DASS-21 has high reliability, a factor structure consistent with its subscales, and high convergent validity with other measures of anxiety and depression [32]. This scale is administered at baseline and 6 months.

##### The Behaviour and Symptom Identification Scale-24

The Behaviour and Symptom Identification Scale-24 (BASIS-24) [33] measures psychiatric symptoms and functional difficulties over the previous week. Items are rated from 0 (no difficulty/symptom never present) to 4 (extreme difficulty/symptom always present). It has adequate reliability (coefficient α from .75 to .91 for subscales), validity, and responsiveness to change (effect size for change=0.56). The scale is administered at baseline and 6 months.

##### Activity and Participation Questionnaire

The Activity and Participation Questionnaire (APQ6) [7] is a measure of the participant’s capacity to conduct their usual activities over the previous month. Consumer feedback and test-retest reliability indicates good construct validity. This questionnaire is administered at baseline, 3 months, and 6 months.

##### The Assessment of Quality of Life-8 Dimension

The Assessment of Quality of Life-8 Dimension (AQoL-8D) [34] is a health-related, multi-attribute, quality-of-life measure, which has 35 items measuring independent living, happiness, mental health, coping, relationships, self-worth, pain, and senses over the previous week. Results indicate it has strong validity, reliability, convergent validity, and predictive validity compared to other measures of quality of life. This measure is administered at baseline and 6 months.

##### The Pittsburgh Sleep Quality Index

The Pittsburgh Sleep Quality Index (PSQI) [35] is a 19-item self-report measure of sleep quality and disturbances over the previous 7 days. The PSQI was used to measure seven domains: subjective sleep quality, sleep latency, sleep duration, habitual sleep efficiency, sleep disturbances, use of sleep medications, and daytime dysfunction. The domains are rated from 0 (no difficulty) to 3 (severe difficulty). A PSQI score above 5 yielded a diagnostic sensitivity of 89.6% and specificity of 86.5% (κ=0.75, *P*<.001) in distinguishing between good and poor sleepers. Generally, good psychometric properties have been established for the PSQI, with acceptable internal homogeneity, test-retest reliability, and validity. This measure is administered at baseline and 6 months.

##### The Working Alliance Inventory–Short Form Revised

The Working Alliance Inventory-Short Form Revised (WAI-SR) [36] measures therapeutic alliance, assessing agreement on the tasks of therapy, goals of therapy, and development of an affective bond. For example, item 1 asks “As a result of these sessions, I am clearer as to how I might be able to change.” The WAI–Short Form Revised (WAI-SR) demonstrated good psychometric properties in an initial validation, with internal reliability (α>.80) and convergent validity with related measures being good [37]. This measure is administered at baseline and 6 months.

#### Other Measurements

##### Measurements From the Embrace2 Device

Participants will have their physiological measurements continuously recorded, including EDA, actigraphy, and temperature, as described in the Interventions section. Actigraphy can be converted into resting time (ie, an approximation of sleep) and step counts. Reports of physiological measures from Empatica are in the form of CSV (comma-separated values) files that can be converted into dashboards and visual files using Splunk. Data transmitted to the research portal can also be examined for participant adherence to the device.

##### Ecological Momentary Assessment

Participants in the integrated mHealth intervention will be asked to report their stress levels in real time to identify periods when they are stressed or becoming unwell. Participants will receive a text message asking, “How stressed are you feeling now?” to be answered on a scale from 0 (not at all stressed) to 10 (very stressed), what they are doing, and/or how they are feeling. Participants will not respond to the text message, rather they will record their response on the Empatica Mate App, which is downloaded onto their phone. Text messages will be sent both according to an automated schedule and manually by the research psychologist. The event will be given a time stamp and recorded alongside participants’ physiological data.

##### Qualitative Interviews Regarding Attitudes Toward the unWIRED Intervention

Feedback will be obtained from participants and their case managers as to the acceptability and usefulness of the approach. For example, participants and case managers will be asked what they found helpful or challenging about using the mHealth device. This information will be sought either from focus groups or individual interviews. The transcribed responses will be thematically analyzed according to Braun and Clarke [38] by members of the research team.

##### Case Manager Report

A monthly report of participant engagement and use of the device will be recorded by the case manager.

### Outline of Study Processes

The following is an outline of the major components in administering the trial: recruitment, consent and initial assessment, administration, and trial conclusion.

#### Recruitment

Recruitment will primarily be managed by the research psychologist, who will explain and provide information to case managers and service managers at the clinical sites. Case managers will ask potential participants if they are interested in being involved. If they indicate interest, the research psychologist will contact them and answer any questions they may have regarding the project.

#### Consent, Randomization, and Initial Assessment

If a participant indicates interest in being involved, the research psychologist will ask for their written consent. Randomization will be determined by an external party who will communicate treatment condition to research staff. After consent and randomization, participants will be asked to complete a series of self-report questionnaires (see the Participant Self-Report, With Time Point, section). The psychologist will ask all participants to download the Empatica Mate App onto their phone and they will set up an Empatica account using a QR (Quick Response) code from the research portal. The Embrace2 device will be paired with the participant’s phone using Bluetooth. They will give the participant some basic instructions on how to operate the device, including how to charge it and keep it connected.

#### Trial Administration

Data will be collected from the Embrace2 in a continuous and ongoing manner for all participants. Case management and treatment will continue as per usual in the community mental health services. Case managers of participants in the integrated unWIRED arm will receive training on how to use the mHealth device and dashboard to monitor a participant’s mental state. Research staff will monitor the extent to which there are data uploaded from the device based on the Empatica Research Portal. If unWIRED participants are observed to have minimal adherence over consecutive days, the research psychologist can contact each participant to determine the reason for the disconnection and attempt to restore the device. During the trial, the research psychologist may send EMA text messages to the unWIRED participants to determine stress levels and their responses will be visible on the Empatica Research Portal. EMA messages will be sent according to a schedule and can also be sent manually by the research psychologist. Physiological data from each unWIRED participant’s previous day will be displayed on the Splunk dashboard, showing their activity, rest, and EDA. A link to the dashboard will be provided to case managers so they can view the information on their work computer. Case managers will use the information from the dashboard to enhance their clinical decisions. Clinical staff and participants will be encouraged to discuss data recorded from the device during their meetings and to include this information in their collaborative care plan. Case managers will be provided with a basic manual for interpreting the dashboard; however, a clinical response will be left to their clinical judgement. Throughout the trial administration, clinical staff will be asked to report on their participant’s ongoing functioning and distress, as described in the Assessments of Mental Deterioration section. The research team can provide support to case managers as needed.

#### Trial Conclusion

**Figure 2:**
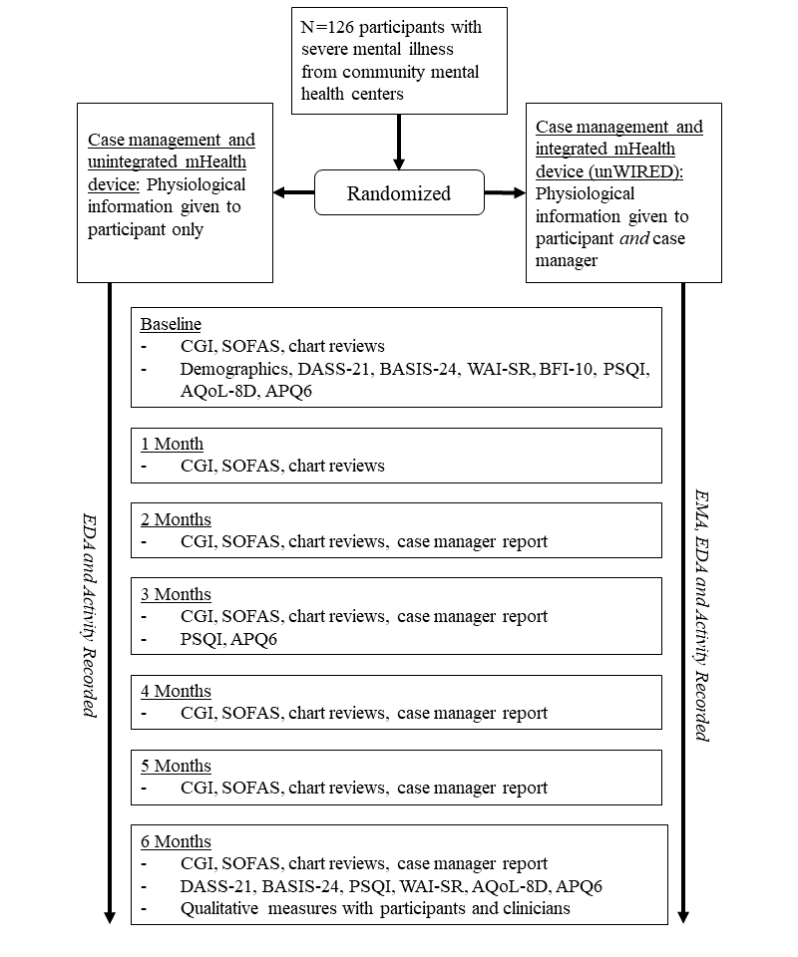
Outline of measurements for the randomized controlled trial. APQ6: Activity and Participation Questionnaire; AQoL-8D: Assessment of Quality of Life-8 Dimension; BASIS-24: Behaviour and Symptom Identification Scale-24; BFI-10: 10-item Big Five Inventory; CGI: Clinical Global Impression Scale; DASS-21: 21-item Depression Anxiety and Stress Scale; EDA: electrodermal activity; EMA: ecological momentary assessment; mHealth: mobile health; PSQI: Pittsburgh Sleep Quality Index; SOFAS: Social and Occupational Functioning Assessment Scale; WAI-SR: Working Alliance Inventory–Short Form Revised.

After the 6-month enrollment in the study, or earlier if a participant asks to withdraw, all participants will once again be asked complete the self-report questionnaires that were administered during the initial appointment (see Participant Self-Report, With Time Point, section). Researchers will ask participants to remove their devices and return them to administrative staff at their clinical site. Each participant will be registered as having “completed” the trial on the Empatica portal, and clinical staff will be informed that they are no longer in the trial. Qualitative interviews may be used after the trial to examine both participant and case manager experiences with the integrated mHealth device. See Figure 2 for the RCT CONSORT-EHEALTH flow diagram.

### Safety Monitoring

This study will be overseen by senior psychiatrists (AH, BK, and JS) who have extensive clinical experience in youth mental health. The participants will have case managers who are mental health professionals working with them on a weekly basis. Adverse events can be reported by either the participants or their case managers and reviewed by an investigational team. Participants will be prompted to report any adverse events. All adverse events will be recorded in the adverse event log in the participant case report form, including the seriousness, severity, relationship to study product, duration, and outcome. Steps will be taken according to the adverse reaction decision tree. In all cases, researchers will maintain contact with participants who experience an adverse event until it has been resolved and symptoms disappear. If required, they will also be asked to notify their general practitioner or primary care doctor. Standard Operating Procedures will be in place to insure the trial is conducted in accordance with Good Clinical Practice. The project will have an independent data safety monitoring board (DSMB) composed of health professionals, researchers, and IT experts who will meet every 4 months. The project will have regular reviews and meetings to determine the safety and integrity of the data.

### Data Collection, Use, and Privacy

Data will be collected by the research team and will be coded so that they will not be identifiable to anyone other than the research team. Data will be collected manually through self-report, interview notes, and EMAs and automatically through the mHealth device. The physiological data will be transmitted to the Empatica interface and downloaded to the project system within a day of recording. However, all data going to Empatica will be deidentified and coded. All data, once converted to electronic format where applicable, will be backed up on a password-protected computer hard drive. Source documents will be kept in participant files, which will be stored in a locked cabinet in a locked room. Data from measures will be entered into a password-protected electronic database on a secure health network drive and backed up onto a password-protected University of Sydney system. No uncoded or identifiable data will be stored outside of the hospital research site. A DSMB, composed of researchers in eHealth, psychiatrists, and information officers will meet to discuss the data management processes associated with this study.

Physiological data will be uploaded onto the secure website of Empatica Inc via its website in the United States. Data will then be retrieved and downloaded onto a University of Sydney server and analyzed within the secure university environment. Information will be returned to treating clinicians operating within NSW Health firewalls. The initial data are controlled by Empatica; they will then be transferred to University of Sydney and ESE (Engineering Und Software-Entwicklung GmbH) servers, which are assisting with data management. Data flow through servers domiciled in either the United States or the European Union is allowed in NSW Health [39] and does not breach the NSW Health Records and Information Privacy Act 2002 [40].

### Power Calculations and Statistical Analysis

The primary outcome for this study is to compare changes in treatment-related outcomes before and after the intervention for participants enrolled in the integrated versus unintegrated conditions. There are currently no trials examining the augmentation of treatment with an mHealth device, so sample size estimations are made using general calculations. Assuming a moderately sized advantage of the integrated (ie, unWIRED) versus unintegrated device on outcomes (effect size=0.5, 80% power, and a 2-tailed α of .05), we will require 63 participants per group.

The RCT component generally compares measures of mental health, distress, and functioning between the conditions, adjusted for baseline scores. For example, this analysis may focus on an intent to treat using a hierarchical linear model (HLM) to test the differential effects of the two treatment conditions, because this method allows the number of observations to vary between participants and handles missing data by calculating estimates of trajectories using maximum likelihood estimation. Fixed effects could be tested for intervention condition and time of assessment. Random effects in the unstructured models provide an index of the relative effects of the treatments over time. Fixed effects parameters may be tested with the Wald test (*t* test, *P*<.05, 2-sided) and 95% confidence intervals. Analyses will focus on the primary outcomes (ie, CGI scores) and secondary outcomes (eg, APQ6, BASIS-24, DASS-21, PSQI, and AQoL-8D) between the unWIRED group and the active control group, with the main outcome points being at the 6-month follow-up relative to baseline. All results are based on estimated mean values derived from HLM analyses. The within-subjects analysis will be comprised of linear fixed effects models using physiological measurements (ie, sleep, activity, and EDA) to predict measures of mental health, distress, and functioning. Baseline variables may be used as covariates when analyzing individual and between-group differences (eg, age, illness severity, treatments, and personality differences). Thematic analysis [38] will be used to examine individual and group qualitative feedback regarding the acceptability of the mHealth device and the unWIRED intervention for participants and case managers; there will be approximately 10-20 participants in the patient and clinician groups.

## Results

The study received scientific and ethical approval at Western Sydney Local Health District. It is due to start in October 2020 and conclude in October 2022.

## Discussion

This study will determine whether integrating an mHealth device into clinical care is useful and effective in managing severe mental illness in young adults in the community. We plan to test this approach in a frontline community mental health setting, where we can examine its feasibility, acceptability, and efficacy for patients and case managers. While previous studies use mHealth devices for participants to self-monitor, this is among the first to stream information to both a patient and the treating mental health team. We hypothesize that the device will identify periods of increased stress and risk of mental deterioration.

A strength of this design is that it examines both between- and within-subjects components. An RCT is a gold standard in determining treatment efficacy, and the within-subjects design can control for individual differences. This study also takes several types of measurements regarding evidence of mental deterioration, symptoms, and functioning. Potential limitations with this protocol should also be noted. This study intends on using participants with a variety of diagnoses, with varying severities, and with psychosocial difficulties. While this increases its ecological validity, the variation may make it more difficult to detect group differences. This limitation highlights the importance of controlling for relevant variables as well as studying within-subjects variation. Furthermore, even severely unwell patients are more often stable than deteriorating, providing fewer opportunities to test the efficacy of the unWIRED approach. Hence, examining process variables is important, as is the mixed methods approach employed in this study. Finally, the design incorporates several measures to detect an effect; however, this increases the likelihood of type I error.

While ambitious, this project’s clinical implications are potentially significant. Any treatment that improves collaboration and early intervention may have a far-reaching impact on the treatment of severe mental illness in the community. The intervention could allow clinicians to remotely and unobtrusively monitor severely unwell patients in the community and provide an effective means to augment treatment.

## Appendix

Multimedia Appendix 1 Randomized controlled trial measurements, measurement reporter, and time points.

Multimedia Appendix 2 Scientific approval and reviewer reports from the authors' institution.

## Abbreviations

ANZCTR: Australian New Zealand Clinical Trials Registry
APQ6: Activity and Participation Questionnaire
AQoL-8D: Assessment of Quality of Life-8 Dimension
BASIS-24: Behaviour and Symptom Identification Scale-24
BFI-10: 10-item Big Five Inventory
CGI: Clinical Global Impression Scale
CONSORT-EHEALTH: Consolidated Standards of Reporting Trials of Electronic and Mobile Health Applications and Online Telehealth
CSV: comma-separated values
DASS-21: 21-item Depression Anxiety and Stress Scale
DSMB: data safety monitoring board
EDA: electrodermal activity
EMA: ecological momentary assessment
ESE: Engineering Und Software-Entwicklung GmbH
HLM: hierarchical linear model
mHealth: mobile health
NSW: New South Wales
PSQI: Pittsburgh Sleep Quality Index
QR: Quick Response
RCT: randomized controlled trial
SOFAS: Social and Occupational Functioning Assessment Scale
WAI: Working Alliance Inventory
WAI-SR: Working Alliance Inventory–Short Form Revised
