# Supplementary material for: Integrating a Mobile Health Device Into a Community Youth Mental Health Team to Manage Severe Mental Illness: Protocol for a Randomized Controlled Trial
Source: JMIR Res Protoc. 2020 Nov 2;9(11):e19510. doi: 10.2196/19510 (PMC7669449; doi:10.2196/19510)
Supplement: Multimedia Appendix 1 [file resprot_v9i11e19510_app1.docx]

Table MA1-1. Randomized controlled trial measurements, measurement reporter and time-points.

| Measures | Description | Reporter | Baseline | 1-month | 2-month | 3-month | 4-month | 5-month | 6-month |
| --- | --- | --- | --- | --- | --- | --- | --- | --- | --- |
| **Mental Deterioration** |  |  |  |  |  |  |  |  |  |
| Clinical Global Impression Scale | Brief measure of symptom severity and treatment response | Clinician +  Blind rater | × | × | × | × | × | × | × |
| Charts reviews | Examined for diagnosis, contacts, hospitalizations, relapse etc. | Clinician +  Blind rater | × | × | × | × | × | × | × |
| Social and Occupational Functioning Assessment Scale | Overall measure of social and occupational functioning | Clinician +  Blind rater | × | × | × | × | × | × | × |
| **Personal** |  |  |  |  |  |  |  |  |  |
| Demographics | Gender, date of birth and ethnicity | Participant | × |  |  |  |  |  |  |
| Big Five Inventory-10 Items | Personality traits openness, conscientiousness, neuroticism, extraversion and agreeableness | Participant | × |  |  |  |  |  |  |

| **Symptoms** |  |  |  |  |  |  |  |  |  |
| --- | --- | --- | --- | --- | --- | --- | --- | --- | --- |
| Depression, Anxiety, Stress Scale 21 items | Current depression, anxiety and stress | Participant | × |  |  |  |  |  | × |
| The Behavior and Symptom Assessment Scale 24 items | Psychiatric symptoms and functioning difficulties | Participant | × |  |  |  |  |  | × |
| Pittsburgh Sleep Quality Index | Sleep quality and disturbances. | Participant | × |  |  | × |  |  | × |
| **Quality of Life** |  |  |  |  |  |  |  |  |  |
| Activity and Participation Questionnaire | Capacity to conduct usual activities | Participant | × |  |  | × |  |  | × |
| Assessment of Quality of Life 8 Dimensions | Health-related multi-attribute measure of quality of life | Participant | × |  |  |  |  |  | × |
| **Therapeutic Alliance** |  |  |  |  |  |  |  |  |  |
| Working Alliance Inventory – Short Form Revised | Working alliance with clinician | Participant | × |  |  |  |  |  | × |
| Case manager report | Report of participant engagement and use of mHealth device | Clinician |  | × | × | × | × | × | × |

| **E2 Measures** |  |  | |  | | | | | | |
| --- | --- | --- | --- | --- | --- | --- | --- | --- | --- | --- |
| Actigraphy | Rest and activity. | Participant | | Continuously and automatically | | | | | | |
| Electrodermal Activity  Adherence | Arousal via electrical properties of skin e.g., non-specific skin conductance.  Number of days and hours recording. | Participant  Participant | | Continuously and automatically  Continuously and automatically | | | | | | |
| **Stress and activities** |  | |  |  | | | | | | |
| Ecological Momentary  Assessment | Participant stress, activity and emotion in real time. | | Participant | Throughout trial | | | | | | |
| **Acceptability** |  | |  |  |  |  |  |  |  |  |
| Qualitative interviews | Participant and case manager interview regarding acceptability of treatment. | | Clinician + Participant |  |  |  |  |  |  | × |

×: Time point of measurement.
