# Supplementary material for: Integrating a Mobile Health Device Into a Community Youth Mental Health Team to Manage Severe Mental Illness: Protocol for a Randomized Controlled Trial
Source: JMIR Res Protoc. 2020 Nov 2;9(11):e19510. doi: 10.2196/19510 (PMC7669449; doi:10.2196/19510)
Supplement: Multimedia Appendix 2 [file resprot_v9i11e19510_app2.pdf]

NSW GOVERNMENT | Health Western Sydney Local Health District

HREC Committee Secretariat:

Dr Tony Skapetis
Dental Graduate

Mrs Patricia Fa
Clinical Trials Pharmacist

Mrs Seema Manoj
Minutes Secretary

HREC Committee Members:

Dr Grahame Ctercteko
Medical Graduate – Colorectal Surgeon

Mr John Fisher
Lawyer

Mr John McLeod
Layperson

Mr Sean Mungovan
Physiotherapist

Dr Christopher Ryan
Medical Graduate - Psychiatrist

Mrs Katherine Schaffarczyk
Nurse Educator

Prof Ramon Shaban
Nursing – Community Health

Dr Howard Smith
Medical Graduate – Endocrinologist

Ms Jennifer Sullivan
Layperson

Dr Christine Wearne
Clinical Psychologist

Ms Sarah Melov
Clinical Midwife Consultant

Prof Vicki Flood
Allied Health

Ms Elizabeth Tran
Investigational Drug Pharmacist

Research Office File No: **(6291)**

Ethics Ref: 2019/ETH13581
Governance Ref:

19 December 2019

Prof Anthony Harris
Department of Psychiatry
Westmead Hospital

Dear Prof Harris

Project title: Use of an integrated mobile health device to manage severe mental illness in young adults in the community: A randomized controlled trial of unwired

Thank you for submitting the above project which was considered by the WSLHD Human Research Ethics Committee at its meeting held on 10 December 2019.

In order to make a determination of the ethical acceptability of your project, please respond to the following request for additional information / clarification:

Scientific Advisory Committee :

Reviewer 1

- The primary outcome measure is change in CGI (Clinical Global Impression) score by "case managers and clinical staff". This is not appropriate:
  - The study is unblinded so scoring may be biased 2
  - The CGI may have poor inter-rater reliability
  - Each component of the CGI is rated separately; the instrument does not yield a global score.
- It is possible that the intervention might be harmful – although there will be clinical oversight by the investigators, the study should have an independent data and safety monitoring board.

Reviewer 2

- This is quite a complex study although the clinical aim of improving detection of early signs of relapse of psychosis is worthwhile and the hypotheses and research questions are credible.
- The methodology may be difficult for some patients to comphrend. While orientated to stress and consideration of stress between patient and case manager, it is possible that data would also register bodily changes in relation to other activities that involve arousal or changes to autonomic function (e.g. sexual and excretory functions) that most people would consider private matters.
- The study does give patients the power to turn off devices when they choose. This area is not specially addressed in the protocol. The protocol and participant information sheet do address issues of privacy and do so in

compliance with legal requirements although there may be limits to the security of digital platforms – however I think privacy can be effectively maintained according to this protocol.

- It was a little unclear as to whether date would be shared with the 'clinical team' – the information suggests it would be only available to patients, clinician and research team. However, if a decision was made involving early intervention, I wonder whether this would require communication about the data to the other members of the team
- Possibly some consideration could be given to details mentioned above. Howvever, overall I conbsider the study to be sound with at least a reasonable prospect of beneficial outcomes. There is a pilot study that was referred to – I couldn't see any details about the outcomes of the pilot study and whether they supported the hypotheses proposed in the new study (perhaps this could be made clearer)

Human Research Ethics Committee

GENERAL: (Ref: National Statement 1.1, 1.4, 1.6)

- The issues identified by SAC should be adequately addressed
- Case Managers will participate in Qualitative interviews however no PICF has been submitted. The researcher should submit a PICF Case Managers for review
- On page 9 of 12 of the Protocol states that "Audio recorded data from interviews will be transcribed." Who will transcribe interviews? If audio transcription is performed by an external company, a signed confidentiality agreement must be submitted. This should be explained in the Protocol and PICF.
- Who is monitoring responses?

In order to facilitate the HREC's reconsideration of your project, please provide the requested information as soon as possible. Your response should be directed to The Secretary, WSLHD Human Research Ethics Committee, Research Office.

Please note that if the requested information is not received within 60 days the application will be cancelled and returned to you.

Should you have any queries about your project, please contact Kellie Hansen, HREC Executive Officer on 8890 9007.

Yours sincerely

Mrs Pat Fa
Secretary
WSLHD Human Research Ethics Committee
